# Gut Microbiota and Metabolic Dysregulation in Elderly Diabetic Patients: Is There a Gender-Specific Effect

**DOI:** 10.3390/jcm14093103

**Published:** 2025-04-30

**Authors:** Magdalena Piłot, Sylwia Dzięgielewska-Gęsiak, Katarzyna Weronika Walkiewicz, Martyna Bednarczyk, Dariusz Waniczek, Małgorzata Muc-Wierzgoń

**Affiliations:** 1 Department of Internal Diseases Propaedeutics and Emergency Medicine, Faculty of Public Health in Bytom, Medical University of Silesia in Katowice, Piekarska 18, 44-902 Bytom, Poland; mpilot@sum.edu.pl (M.P.); sgesiak@sum.edu.pl (S.D.-G.); kk.walkiewicz@gmail.com (K.W.W.); 2 Department of Cancer Prevention, Faculty of Public Health, Medical University of Silesia in Katowice, 40-752 Katowice, Poland; martyna.bednarczyk@outlook.com; 3 Department of Oncological Surgery, Faculty of Medical Sciences in Zabrze, Medical University of Silesia in Katowice, 41-808 Katowice, Poland; dwaniczek@sum.edu.pl

**Keywords:** type 2 diabetes, gut microbiome, sex, carbohydrate disorders lipid disorders

## Abstract

**Background/Objectives:** The aim of this study was to qualitatively and quantitatively assess the bacterial domain of the gut microbiome in elderly patients with type 2 diabetes (T2D), with a focus on sex differences, glycemic control, and lipid disorders. **Methods:** This study included 60 older adults with T2D (38 women and 22 men) treated with metformin or a combination of metformin and insulin. The gut microbiota was profiled using 16S rRNA gene sequencing. Statistical analyses, including correlation analysis and multiple regression, were performed to identify the associations between microbial taxa, sex, and metabolic parameters. **Results:** No statistically significant differences in alpha or beta diversity were observed between the sexes. Multiple regression analysis indicated a positive relationship between *Tenericutes* and HbA1c in male participants (β = 2.22931, CI [0.75, 3.70], R = 0.67; R^2^ = 0.36; unadjusted *p* = 0.0052; adjusted *p* = 0.0496). In female participants, G0′ (β = −2.24107, CI [−3.19, −1.30], R = 0.78; R^2^ = 0.58; unadjusted *p* = 0.00003; adjusted *p* = 0.0005) and HbA1c (β = −1.86670, CI [−2.61, −1.12], R = 0.78; R^2^ = 0.58; unadjusted *p* = 0.00001; adjusted *p* = 0.0003) correlated negatively with *Verrucomicrobia* as well G0′ (β = −1.90427, CI [−2.95, −0.85], R = 0.46; R^2^ = 0.17; unadjusted *p* = 0.0008; adjusted *p* = 0.007) and HbA1c (β = −1.69561, CI [−2.52, −0.87], R = 0.46; R^2^ = 0.17; unadjusted *p* = 0.0002; adjusted *p* = 0.002) correlated negatively with OD1 bacteria, known as *Parcubacteria*. **Conclusions:** In this elderly population with type 2 diabetes, biological sex did not significantly affect the gut microbiota diversity. However, several exploratory associations between microbial taxa and metabolic parameters differed between men and women, suggesting that sex may influence specific aspects of microbiota—metabolism interactions. These preliminary findings underscore the importance of considering both age- and sex-related factors when investigating the gut microbiome in the context of type 2 diabetes.

## 1. Introduction

Diabetes mellitus is among the most prevalent non-communicable diseases (NCDs) and is recognized by the World Health Organization (WHO) as a major global health challenge of the 21st century, alongside cardiovascular diseases, cancer, chronic respiratory diseases, and neurodegenerative disorders. Together, these conditions account for over 70% of global deaths, mainly due to modifiable risk factors such as poor diet, sedentary behavior, smoking, and environmental exposure [1]. The number of people living with diabetes has more than quadrupled since 1990, now exceeding 800 million globally [2]. According to WHO projections, diabetes will be the seventh leading cause of death by 2030 [3].

In recent years, accumulating evidence has linked type 2 diabetes (T2D) to disturbances in the gut microbiome [4,5,6]. The gut microbiota comprises a diverse community of microorganisms residing in the gastrointestinal tract, primarily the colon, and includes bacteria, viruses, fungi, and archaea. These microbes play essential roles in nutrient absorption, vitamin synthesis, immune modulation and energy homeostasis [7,8,9]. Alterations in microbial composition (dysbiosis) have been implicated in the development of insulin resistance, low-grade inflammation, and metabolic disturbances associated with T2D [10,11,12,13].

One of the key mechanisms underlying this relationship is the production of microbial metabolites, such as short-chain fatty acids (SCFAs), including butyrate and propionate, which promote insulin sensitivity and regulate glucose metabolism [14]. In individuals with T2D, SCFA production is often diminished, while the increased translocation of lipopolysaccharides (LPS) from Gram-negative bacteria into the bloodstream—a process known as metabolic endotoxemia—triggers systemic inflammation and exacerbates insulin resistance [15,16].

Several specific bacterial taxa have been implicated in these processes. A decrease in beneficial butyrate-producing genera, such as *Faecalibacterium*, *Roseburia*, and *Eubacterium,* has been consistently observed in individuals with T2D. Conversely, an increased abundance of opportunistic or pro-inflammatory bacteria, including *Ruminococcus gnavus*, *Desulfovibrio*, and certain *Proteobacteria* (e.g., *Escherichia* spp.), has been associated with enhanced LPS production, low-grade inflammation, and impaired glucose metabolism [17,18,19,20].

Importantly, the composition and function of the gut microbiome are influenced by multiple factors, such as age, sex, comorbidities, medication use, lifestyle, stress, and diet. While the microbiota is relatively stable in healthy adults, various internal and external factors can lead to temporary or long-term disruptions in microbial diversity and abundance [21,22,23,24,25]. In this context, biological sex has emerged as an important but underexplored determinant of gut microbiota composition and its implications for disease development.

Both animal and human studies have demonstrated sex-related differences in the gut microbiota, likely mediated by sex hormones and immune responses [26,27,28,29,30]. The term *microgenderome* has been introduced to describe the bidirectional interaction between sex hormones and the gut microbiota. Estrogens and androgens influence the abundance and activity of specific bacterial taxa, and conversely, the microbiota can modulate sex hormone metabolism, potentially impacting disease risk and progression.

Women generally display greater microbial diversity than men, and the bacteria-to-human cell ratio has been estimated to be 2.2 in women versus 1.3 in men [27]. Estrogen has been linked to higher levels of beneficial bacteria, such as *Akkermansia muciniphila* and *Faecalibacterium prausnitzii*, which are known for their anti-inflammatory and barrier-preserving properties, especially in premenopausal women [30,31]. Conversely, men tend to harbor a higher abundance of pro-inflammatory bacteria, which may predispose them to metabolic inflammation and insulin resistance [29].

Studies suggest that testosterone is associated with less diverse microbiota. Low testosterone levels, common in aging men or those with metabolic syndrome, have been linked to a dysbiotic gut profile and an increased risk of T2D [32]. Such dysbiosis is characterized by a higher abundance of opportunistic pathogens and Gram-negative bacteria, including *Lachnoclostridium*, *Blautia*, and *Bergeyella*.

Moreover, sex-specific differences in microbiota responses to dietary interventions have been observed. For instance, Zhao et al. [33] showed that a high-fiber diet led to greater increases in SCFA-producing bacteria in women than in men, resulting in better glycemic control and metabolic improvement in women.

Aging further modulates the gut microbiota, with both men and women showing a reduction in microbial diversity and functional capacity [34]. Whether these changes are predominantly driven by declining sex hormones or by other age-related factors, such as comorbidities, medications, reduced social contact, and dietary patterns, remains unclear. Nonetheless, the interaction between sex, aging, and metabolic health may contribute to distinct microbiota profiles in older adults.

Given the increasing prevalence of T2D in older populations and the growing recognition of sex-related differences in the microbiome, further research is warranted to better understand these interactions. The aim of the present study was to qualitatively and quantitatively assess the composition of the gut bacterial microbiota in elderly patients with type 2 diabetes, with a particular focus on sex differences, glycemic control, and lipid metabolism disorders.

## 2. Materials and Methods

### 2.1. Materials

#### 2.1.1. Ethics

The study protocol was registered with the Bioethical Committee of the Medical University of Silesia in Katowice. The Committee stated that “the project does not meet the criteria of a medical experiment under the applicable law and does not require assessment by the bioethical committee” (decision no. KNW/0022/KB1/39/19). In accordance with this decision, written informed consent for participation was not required, nor was separate patient consent necessary for statistical analyses or for research purposes.

Nonetheless, in line with the principles of the Declaration of Helsinki (2013), participation in the study was entirely voluntary and based on informed decision-making. All participants were provided with comprehensive information regarding the study prior to their inclusion. To safeguard personal data, pseudonymization was applied, meaning that identifying information was processed in such a way that individuals could not be identified without access to a separate re-identification key.

#### 2.1.2. Study Group

The study initially included 358 patients with type 2 diabetes who visited the Diabetes Outpatient Clinic. For the final analysis, 60 individuals (38 women and 22 men) aged between 59.5 and 72.0 years (median 64.0 years) who had diabetes for 5 to 10 years were qualified as meeting all the established criteria.

#### 2.1.3. Inclusion Criteria

Metformin monotherapy (administered at an effective dose to achieve a therapeutic effect) or combined therapy with metformin and insulin (both at effective doses). In both subgroups, treatment was administered for at least 6 months prior to inclusion in the study.

#### 2.1.4. Exclusion Criteria

The exclusion criteria were as follows: patients using hypoglycemic agents other than metformin or a combination of metformin and insulin; those treated within the last month with antibiotics (including antifungals), nonsteroidal anti-inflammatory drugs (NSAIDs), metamizole, paracetamol, corticosteroids, iron supplements, drugs used for gastrointestinal disorders (e.g., proton pump inhibitors), or medications affecting bowel motility or frequency; chronic users of prebiotics, probiotics, or synbiotics; individuals with diagnosed and treated chronic gastrointestinal diseases; patients with coexisting conditions such as cancer, uncompensated heart failure, bronchial asthma, Lyme disease, end-stage renal failure, liver cirrhosis, autoimmune diseases, or endocrine disorders (e.g., hyper- or hypothyroidism); individuals who smoked or abused alcohol; and those who did not provide informed consent to participate in the study.

All patients enrolled in the study received structured education from physicians, diabetes educators, and dietitians. In accordance with current recommendations, both individualized sessions and group educational programs (comprising 6–10 participants) were conducted concurrently. The educational content included comprehensive information on the symptoms and management of diabetes, dietary guidelines, and the role of physical activity in the control of the disease.

### 2.2. Methods

#### 2.2.1. 3-Day Nutritional Interview

Dietary intake was assessed using a three-day food record, which included two weekdays and one weekend day. Prior to data collection, all participants received standardized training from a qualified dietitian on accurate portion size estimation. During training, an Album of Photographs of Food Products and Dishes was used as a visual aid [35].

Each food record was individually reviewed and verified by a dietitian in the presence of the participant to ensure completeness and accuracy. Dietary data were analyzed using the Dietetyk 2.0 software (JuMar, Poznań, Poland), which is based on Polish food composition tables developed by the National Institute of Food and Nutrition. The program enables the analysis of energy, macro- and micronutrient content in the diet and allows for a comparison with dietary reference values [36].

Nutrient intake in the study population was reported as mean values, standard deviations, and medians. Dietary sources of protein, fat, and carbohydrates were expressed as the percentage contribution of each food source to the total intake of the respective macronutrients.

#### 2.2.2. Blood Sample Collection

Blood samples were taken as a routine check-up of biochemical parameters, which was ordered for a patient presenting for a scheduled appointment with a diabetologist. Blood samples for the tests were collected in the morning, on an empty stomach, at a certified hospital-based laboratory, where biochemical analyses were also performed according to an established procedure. The panel of laboratory tests included—fasting plasma glucose (G0′) concentration, glycated hemoglobin (HbA1c) concentration, classical self-monitoring of glycemia (Glu), lipid measurements (concentration of total plasma cholesterol (TC), high-density lipoproteins cholesterol (HDL-C), and triglycerides (TAG), fasting creatinine (Cre) concentration and an estimated Glomerular Filtration Rate (eGFR) was calculated by the Modified Diet in Renal Disease formula (MDRD).

#### 2.2.3. Stool Sample Collection

Stool samples (2 g) were collected in sterile containers and delivered to the laboratory within two hours, where they were frozen at −80 °C before being transferred to a molecular laboratory for analysis.

#### 2.2.4. Qualitative and Quantitative Analysis of the Intestinal Microbiome

Quantitative and qualitative examinations of the fecal intestinal microbiota were performed using next-generation sequencing (NGS) by A&A Biotechnology (Gdynia, Poland) in collaboration with Macrogene, Inc. (Seoul, Republic of Korea) based on the methodology described by Klindworth et al. [37,38]. Taxonomic profiling was carried out at multiple taxonomic ranks, from phylum (L2) to species level, to ensure high-resolution characterization of the microbial community structure.

The analysis was conducted in the following stages:

1. Isolation of DNA from frozen human stool samples

Total DNA was extracted from frozen fecal material using a protocol combining mechanical and enzymatic lysis, followed by purification using ion-exchange membranes. The resulting DNA eluates were required to meet a minimum concentration of 0.1 ng/μL in a volume of no less than 20 μL per sample to ensure their suitability for downstream applications.

2. Amplicon library preparation and sequencing using Illumina SBS technology

Amplicon libraries were constructed by PCR amplification of the V3–V4 hypervariable region of the 16S rRNA gene using a specific primer set with simultaneous incorporation of Illumina adapter sequences and dual indexing barcodes. To ensure data comparability and methodological consistency, library preparation and sequencing were performed according to the protocol described by Klindworth et al. [37].

3. Assessment of DNA and library quality

The quality and quantity of the input DNA were evaluated using agarose gel electrophoresis and fluorometric quantification (Victor fluorometer, PicoGreen assay). The integrity and concentration of the final libraries were assessed to verify their suitability for sequencing.

4. High-throughput sequencing

Sequencing was performed on the Illumina MiSeq platform (v2.6, Illumina, San Diego, CA, USA) using the MiSeq Reagent Kit v3 chemistry in a 2 × 300 bp paired-end format (600 cycles). Libraries were sequenced to a depth of up to 100,000 reads per sample using proprietary reagents, including Herculase II Fusion DNA Polymerase (Agilent Technologies, Santa Clara, CA, USA) and Nextera XT Index Kit v2 (Illumina, San Diego, CA, USA).

5. Bioinformatic analysis

Taxonomic and statistical analyses of the resulting metagenomic data were conducted using the open-source software Statistical Analysis of Metagenomic Profiles (STAMP, version 2.1.3) in accordance with the methodology outlined by Klindworth et al. [37].

Taxonomic summaries were performed at the phylum (L2), class (L3), order (L4), family (L5), genus (L6), and species levels using QIIME 2, allowing for a comprehensive characterization of microbial diversity within the samples [39,40,41].

#### 2.2.5. Statistical Analysis

##### Biochemical Analysis

Statistical analyses were performed using Statistica (version 13.3) for Windows. In our study, we initially included a large cohort of 358 patients with type 2 diabetes. However, after applying the inclusion and exclusion criteria, we finalized our analysis with a sample size of 60 patients (38 women and 22 men). We conducted a post-hoc power analysis to assess the adequacy of the sample size. In the context of power analysis, we adopted a significance level of α = 0.05 and a statistical power of 80%.

The Shapiro–Wilk test was applied to assess the normality of the variable distributions in the participants (all subjects in the study, men and women). As most of the data did not follow a normal distribution, a nonparametric Mann–Whitney U test was used to evaluate the differences between males and females.

##### Statistical Relationships Between Microbiological and Clinical Data

Bioinformatic analysis of the microbiome data was conducted using QIIME2 (version 2019.10.0). Statistical differences in the relative abundance of bacterial phyla and orders between groups were assessed using paired Student’s *t*-test (*p* < 0.05). Alpha diversity was evaluated using the Chao1 and Shannon indices, which were calculated using the *phyloseq* package in R. These indices were statistically analyzed using the Mann–Whitney *U* test and Student’s *t*-test. Beta diversity (β-diversity) was estimated using the UniFrac distance metric and visualized using principal coordinate analysis (PCoA) [42,43,44].

The Spearman rank correlation test was used to assess the strength of the association between these variables. The relationship between variables was categorized according to the correlation coefficient value: slight (r < 0.0), weak (0.1 ≤ r < 0.3), moderate (0.3 ≤ r < 0.5), strong (0.5 ≤ r < 0.7), very strong (0.7 ≤ r < 0.9), and nearly perfect (0.9 ≤ r < 1).

In our multivariable regression models, we employed a combination of approaches for variable selection, which included univariable analysis, clinical judgment, and consideration of previously established associations in the literature. Initially, we conducted univariable analyses to assess the relationship between each independent variable (e.g., sex, HbA1c, and plasma lipids) and the dependent variable (gut microbiome taxa). This step allowed us to identify variables that were significantly associated with the outcome. Following the univariable analysis, we incorporated clinical judgment to select variables that were clinically relevant and supported by the existing literature. Finally, we conducted a thorough assessment of the collinearity among the independent variables included in our models. The analyzed models included the following:

(A) Sex, HbA1c, and G0
(B) Gender, T-C, HDL-C, and TAG (LDL-C was excluded as it is a derivative of the analyzed variables).

The results indicated that multicollinearity was not a significant concern, as all variance inflation factor values were below the threshold of 5. This assessment helped ensure that our regression coefficients were reliable and interpretable. To control for multiple comparisons and reduce the risk of false positives, false discovery rate (FDR) correction (Benjamini-Hochberg procedure) was applied to the *p*-values obtained from regression models. This adjustment was performed to account for the multiple tests associated with the simultaneous analysis multiple microbiome taxa and biochemical variables. A *p*-value of less than 0.05 was considered statistically significant after FDR correction.

## 3. Results

### 3.1. Characteristics of the Study Group

The study initially included 358 patients with type 2 diabetes. For the final analysis, we qualified 60 patients aged 59.5–72.0 years (median 64.0 years) who had diabetes for 6 to 10 years, meeting all the planned criteria. This group included 38 women aged 61.0–75.0 years (median 69.5 years) and 22 men aged 55.0–63.0 years (median 60.0 years). The average dose of metformin for all patients was 2000 mg/day. Metformin was included in patients from the moment of diabetes diagnosis. Among those treated with additional insulin, 55% used human insulin (short-acting/intermediate-acting or a mixture), and 45% used analog insulin (fast-acting, long-acting or a mixture in a 30:70 schedule)—Table 1.

In the analyzed group, multimorbidity predominated. Apart from type 2 diabetes, hypertension was the most prevalent chronic condition, affecting 82% of seniors, followed by atherosclerosis (80%). Cardiovascular diseases, such as coronary artery disease (45%), heart failure (22%), and atrial fibrillation (9%), were also highly prevalent. Additionally, chronic obstructive pulmonary disease was present in 16% of the patients. Patients undergoing chronic treatment, most commonly used angiotensin-converting enzyme inhibitors (ACE inhibitors), angiotensin II receptor blockers (ARBs), beta-blockers, thiazide-like or loop diuretics, statins, and long-acting beta-agonists (patients with COPD).

Diabetes compensation was similar in both groups (HbA1c, 7.0% in women vs. 7.4% in men). Of the other biochemical parameters, only serum HDL cholesterol levels differed significantly (*p* = 0.005).

Analysis of 3-day dietary records revealed several statistically significant differences in nutrient intake between the sexes—Table 2.

Men had significantly higher total energy intake (8960.7 kJ vs. 8087.9 kJ, *p* = 0.000125) and caloric intake (2133.5 kcal vs. 1925.7 kcal, *p* = 0.005937). Protein consumption was also higher among men than among women (79.4 g vs. 68.6 g, *p* = 0.000055), as was the intake of saturated fatty acids (36.1 g vs. 25.1 g, *P* = 0.001073) and carbohydrates (299.7 g vs. 244.4 g, *p* = 0.008693).

Dietary fiber intake was similar between the groups, although the values require confirmation due to missing data.

Additionally, the man demonstrated a higher intake of vitamins B6 and C, accompanied by a significantly lower intake of vitamin A (*p* = 0.0139). No significant differences were observed in mineral intake.

### 3.2. Gut Microbiome Analysis

**Figure 1:**
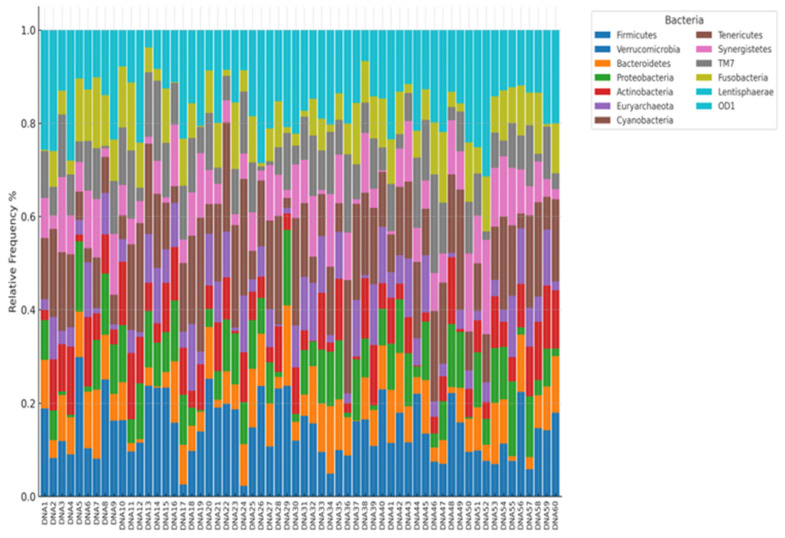
Analysis of biological material (feces) of patients included in the study—taxonomic level type (level–L2). Legend: DNA nr—consecutive patient (according to the order of material collection), variable—abundance and biodiversity of intestinal bacteria.

In the fecal samples, only one domain—Bacteria—was evaluated, as it constitutes the primary component of the human intestinal microbiome. In the analyzed patients with type 2 diabetes mellitus, the predominant bacterial phyla included *Firmicutes* (notably the order *Clostridiales*—47.8% of the total bacterial abundance), *Verrucomicrobia* (*Verrucomicrobiales*—15.78%), followed by *Bacteroidetes* (*Bacteroidales*—8.65%), and *Proteobacteria* (*Enterobacteriales*—8.02%) (Figure 1).

**Figure 2:**
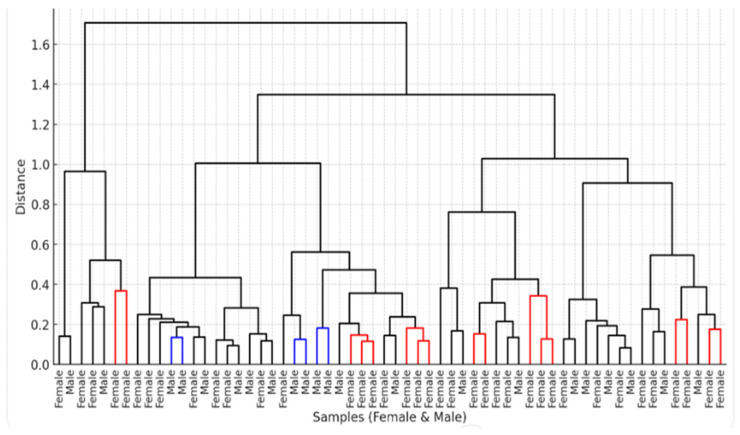
Hierarchical clustering dendrogram (Bray-Curtis Distance) of fecal samples based on their microbiota profiles. The red branches represent female samples. Blue branches indicate male samples. Black branches indicate mixed or unclear groupings, where male and female samples are clustering together.

The hierarchical clustering dendrogram revealed two major clusters, one of which could be further subdivided into distinct subclusters. The first main cluster (one branch of the dendrogram) comprised samples with relatively similar microbial compositions. The second, larger cluster (the opposite branch) displayed greater internal variability and was further divided into smaller subclusters. Notably, male and female samples were intermixed within both clusters, indicating no clear separation by sex (Figure 2).

**Figure 3:**
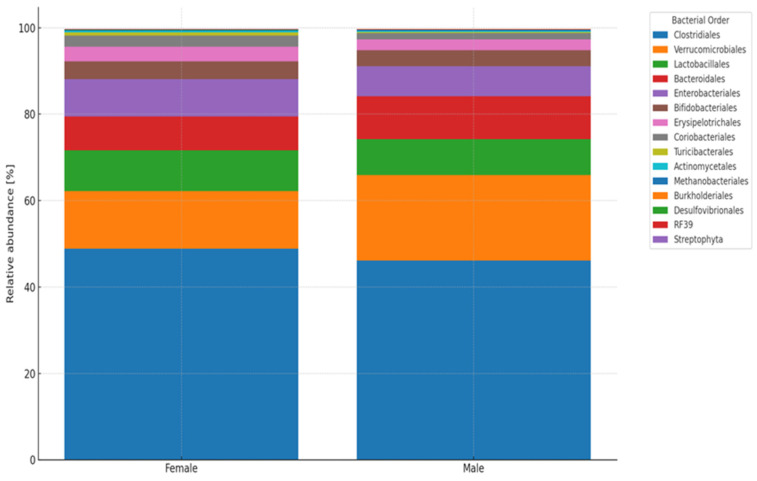
Analysis of biological material (feces) of patients divided by sex—order taxonomic level (L4). Legend: The vertical black line separates the analysis of stool samples of women (**left** side of figure) from those of men (**right** side); variable—abundance and biodiversity of intestinal bacteria.

*Clostridiales* were the most common in the biological material, both in the analysis of the entire study group and in the subgroups of females (48.8%) and males (46.12%)—Figure 3. Then Verrucomicrobiales (F—13.31%; M—19.76%), Lactobacillales (F 9.4%, M 8.3%), Bacteroidales (F—7.87%; M—9.91%), Enterocabacteriales (F—8.65%; M—6.99%). No significant differences by gender.

### 3.3. Alpha and Beta Diversity of the Gut Microbiome

**Figure 4:**
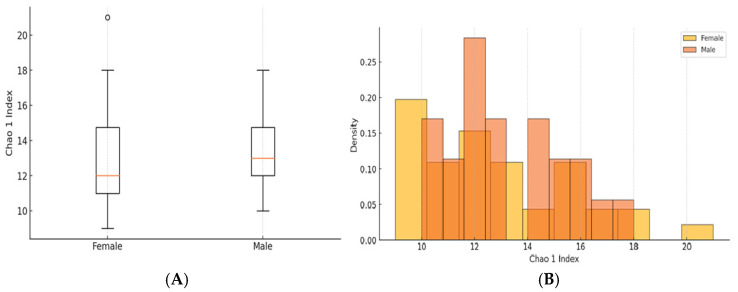
(**A**)—Comparison of Chao 1 Index between females and males: (**B**)—Histogram of Chao 1 Index for both groups.

The Chao1 index estimates species richness by accounting for rare species. In this dataset, there was no strong statistical evidence that gut microbiome diversity differed between sexes (Mann−Whitney U test, *p* = 0.4120; Figure 4). The species compositions within both groups were similar.

**Figure 5:**
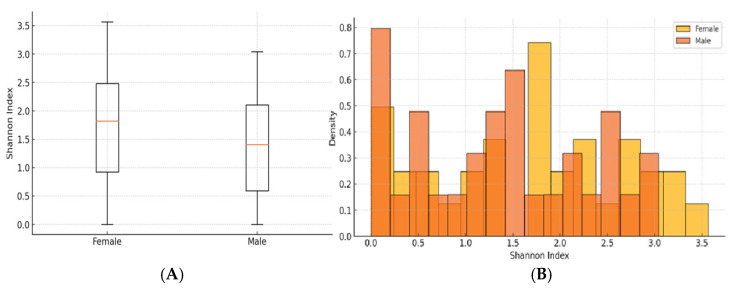
(**A**) Shannon Index distribution across female and male groups—Median (central line in each box), Interquartile range (IQR, the box itself), Potential outliers (dots outside the whiskers); (**B**)—Histogram of the Shannon Index for both—F and M groups.

The results suggest a potential, though not statistically significant, difference in Shannon diversity between the sexes. Specifically, the Mann−Whitney U test yielded a *p*-value of 0.0854, while the parametric *t*-test produced a *p*-value of 0.1991 (Figure 5). These findings may indicate subtle variations in alpha diversity related to sex, which warrants further investigation.

In boththe boxplot and histogram, it was observed that the groups had overlapping distributions, indicating that their microbial diversity was relatively similar.

**Figure 6:**
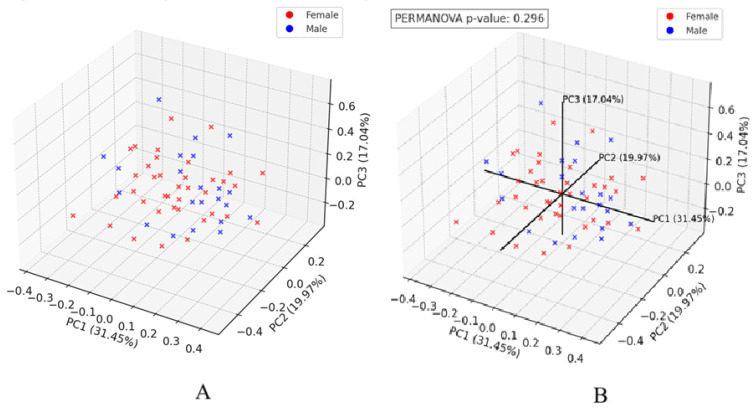
(**A**)—PCoA (Bray-Curtis Dissimilarity) with gender distinction—these three axes capture most of the differences between samples. (**B**)—PCoA (Bray-Curtis Dissimilarity) with sex, axes, and PERMANOVA results.

A multivariate statistical technique—Principal Coordinates Analysis (PCoA), was used to explore and visualize similarities or dissimilarities between samples—Figure 6. Each point represents a microbiome sample, and the distances between points indicate how similar/different they are. The percentage of explained variation for each axis shows how well the axis captures the similarities and differences between samples. The grouping of samples in a graph can suggest common patterns.

Statistical analysis of interindividual variation indicated that the overall taxonomic and functional composition of the gut microbiome did not differ significantly between the sexes. The distribution of female and male samples overlapped substantially, suggesting similar microbial community structures across both sexes.

### 3.4. Correlation Analysis of Microbiological and Clinical Data

Spearman rank correlation analysis revealed several associations between microbiota composition and clinical parameters—Table 3, Table 4 and Table 5.

Glycated hemoglobin (HbA1c) levels showed a positive correlation with the abundance of *Tenericutes* (r = 0.3212) and *Verrucomicrobia* (r = 0.3291). High-density lipoprotein (HDL) cholesterol levels were positively correlated with *Firmicutes* (r = 0.2728), while triglyceride concentrations were negatively correlated with *Tenericutes* (r = –0.2630)—Table 3.

**Table 3:** The linear correlation analysis of the gut microbiome and variables in all patients. Statistically significant correlations are highlighted in gray and red.

	Age	Glu 0′	HbA1c	TC	HDL−C	LDL	TG
k__Bacteria.p__Actinobacteria	0.004366	−0.096310	−0.084351	−0.072133	0.027258	−0.092556	−0.216733
k__Bacteria.p__Bacteroidetes	−0.181849	0.072217	−0.111222	0.133148	−0.174660	0.094360	0.230008
k__Bacteria.p__Cyanobacteria	0.131550	0.065780	0.239915	−0.049634	−0.154532	−0.022733	0.003591
k__Bacteria.p__Firmicutes	0.128621	−0.044541	−0.137082	0.078355	**0.272798**	−0.071186	−0.180217
k__Bacteria.p__Fusobacteria	0.065568	−0.020552	−0.030426	−0.002493	−0.102946	0.091236	−0.166058
k__Bacteria.p__Lentisphaerae	−0.138691	0.005785	0.082054	−0.074258	−0.112869	−0.051308	0.119171
k__Bacteria.p__OD1	0.106919	0.062554	0.040379	−0.165562	−0.073868	−0.188618	0.113417
k__Bacteria.p__Proteobacteria	0.085212	−0.146104	0.022203	−0.226458	−0.059852	−0.178138	−0.119425
k__Bacteria.p__Synergistetes	0.073574	−0.187279	0.250789	−0.196845	−0.244793	−0.112624	−0.218001
k__Bacteria.p__TM7	−0.033153	−0.110339	−0.072516	0.001142	0.164117	−0.123292	0.081520
k__Bacteria.p__Tenericutes	−0.063305	0.106805	**0.321177**	−0.039596	0.064411	−0.042761	**−0.262965**
k__Bacteria.p__Verrucomicrobia	0.107448	0.10843	**0.329106**	−0.035126	−0.233006	0.050656	0.069857

Women—HbA1c value positively correlates with the presence of *Verrucomicrobia*. r = 0.389013, and negatively with HDL-cholesterol, r = −0.359235. Triglyceride concentration negatively correlates with the presence of *Lentisphareace* bacteria. r = −0.338753. Total cholesterol concentration negatively correlates with the presence of *Proteobacteria.* r = −0.477102 and *Synergistetes*, r = −0.333911. LDL cholesterol concentration negatively correlates with the presence of Proteobacteria, r = −0.437380—Table 4.

**Table 4:** The linear correlation analysis of the gut microbiome and variables in females. The statistically significant correlations are highlighted in gray and red.

	Age	Glu 0′	HbA1c	TC	HDL-C	LDL	TG
k__Bacteria.__	0.116224	0.098127	−0.026447	0.143268	0.009665	0.143757	0.234049
k__Bacteria.p__Actinobacteria	−0.117699	−0.120260	−0.025734	0.021337	0.203195	−0.078893	−0.237553
k__Bacteria.p__Bacteroidetes	−0.220822	0.267659	−0.004599	0.224204	−0.102528	0.153737	0.215669
k__Bacteria.p__Cyanobacteria	0.159672	0.226343	0.153605	−0.130926	−0.194769	−0.057843	0.093844
k__Bacteria.p__Firmicutes	0.313096	−0.115117	−0.168090	0.075829	0.163803	0.063574	−0.303863
k__Bacteria.p__Fusobacteria	−0.024158	−0.141379	0.020784	−0.050849	−0.053457	−0.103933	−0.173408
k__Bacteria.p__Lentisphaerae	−0.191739	0.234850	0.011928	0.111180	−0.143956	0.148426	**0.338753**
k__Bacteria.p__OD1	0.032102	0.276792	0.151255	−0.315146	−0.238875	−0.296429	0.178980
k__Bacteria.p__Proteobacteria	−0.001096	−0.291092	0.116410	**−0.477102**	−0.213711	**−0.437380**	−0.052197
k__Bacteria.p__Synergistetes	0.026112	−0.092562	0.251231	**−0.333911**	−0.146191	−0.308584	−0.311191
k__Bacteria.p__TM7	−0.112554	−0.153672	−0.008604	−0.016952	0.155371	−0.125736	−0.000244
k__Bacteria.p__Tenericutes	−0.165330	0.113330	0.286782	0.005175	0.048302	0.038217	−0.307196
k__Bacteria.p__Verrucomicrobia	0.008276	0.246017	**0.389013**	−0.188830	**−0.359235**	−0.065326	0.048799

Men—Glucose concentration negatively correlates with the presence of *Bacteroidetes*, r = −0.428571. Total cholesterol concentration negatively correlates with the presence of *Lentisphaerae* r = −0.494741. HDL cholesterol concentration positively correlates with the presence of *Firmicutes*, r = 0.446640. LDL cholesterol concentration positively correlates with the presence of *Fusobacterium*, r = 0.436091—Table 5. 

**Table 5:** The linear correlation analysis of the gut microbiome and variables in male participants. Statistically significant correlations are highlighted in gray and red.

	Age	Glu 0′	HbA1c	TC	HDL-C	LDL	TG
k__Bacteria.p__Actinobacteria	0.226191	0.051977	−0.168457	−0.300000	−0.288701	−0.156497	−0.259887
k__Bacteria.p__Bacteroidetes	0.049859	**−0.428571**	−0.287006	−0.010728	−0.330322	−0.064935	0.156409
k__Bacteria.p__Cyanobacteria	0.184135	−0.167781	0.351799	0.153594	−0.247970	0.083890	−0.170865
k__Bacteria.p__Firmicutes	−0.077621	0.093168	−0.051977	0.059289	**0.446640**	−0.315641	0.264822
k__Bacteria.p__Fusobacteria	0.109157	0.195815	−0.129671	0.140949	−0.021757	**0.436091**	−0.209059
k__Bacteria.p__Lentisphaerae	−0.080681	−0.409604	0.211070	**−0.494741**	−0.125814	−0.415280	−0.400144
k__Bacteria.p__OD1	0.242992	−0.279060	−0.123992	0.082299	0.243114	−0.038785	0.036893
k__Bacteria.p__Proteobacteria	0.003399	0.199322	−0.158192	0.125918	−0.102202	0.230943	−0.238848
k__Bacteria.p__Synergistetes	0.213852	−0.398563	0.174627	0.107402	−0.317172	0.232425	−0.077195
k__Bacteria.p__TM7	−0.106916	0.050776	−0.168195	−0.064624	−0.012309	−0.156945	**0.426213**
k__Bacteria.p__Tenericutes	0.246659	0.089452	0.388755	−0.148176	0.197341	−0.189147	−0.253333
k__Bacteria.p__Verrucomicrobia	0.345930	−0.169384	0.204289	0.290861	0.102657	0.252080	0.071290

The multiple regression analysis indicated a positive relationship in male participants between *Tenericutes* and G0′ (β = 3.24592, CI [0.46, 6.03], R = 0.67; R^2^ = 0.36; unadjusted *p* = 0.02; adjusted *p* = 0.10) and HbA1c (β = 2.22931, CI [0.75, 3.70], R = 0.67; R^2^ = 0.36; unadjusted *p* = 0.0052; adjusted *p* = 0.0496).

In female participants, G0′ (β = −2.24107, CI [−3.19, −1.30], R = 0.78; R^2^ = 0.58; unadjusted *p* = 0.00003; adjusted *p* = 0.0005) and HbA1c (β = −1.86670, CI [−2.61, −1.12], R = 0.78; R^2^ = 0.58; unadjusted *p* = 0.00001; adjusted *p* = 0.0003) correlated negatively with *Verrucomicrobia* as well G0′ (β  = −1.90427, CI [−2.95, −0.85], R = 0.46; R^2^ = 0.17; unadjusted *p* = 0.0008; adjusted *p* = 0.007) and HbA1c (β = −1.69561, CI [−2.52, −0.87], R = 0.46; R^2^ = 0.17; unadjusted *p* = 0.0002; adjusted *p* = 0.002) correlated negatively with OD1 bacteria, known as *Parcubacteria*—Table 4.

Additionally, multiple regression analysis revealed associations between lipid parameters and microbiota composition in the female participants. Specifically TC (β = −2.64179 CI [−4.44, −0.85], R = 0.74; R^2^ = 0.45; unadjusted *p* = 0.005; adjusted *p* = 0.02) and HDL-C (β = −4.76610 CI [−5.08, −2.10], R = 0.74; R^2^ = 0.45; unadjusted *p* = 0.02; adjusted *p* = 0.04) and TG (β = −5.58098 CI [−5.17, −1.24], R = 0.74; R^2^ = 0.45; unadjusted *p* = 0.0003; adjusted *p* = 0.003) correlated with *Cyanobacteria* and HDL-C (β = 4.864989 CI [4.06, 5.22] R = 0.73; R^2^ = 0.43; unadjusted *p* = 0.02; adjusted *p* = 0.04) correlated with *Fusobacteria*.

## 4. Disscusion

### 4.1. Gender-Specific Microbiota–Metabolism Interactions in Elderly Diabetic Patients

Many human trials have shown that sex can influence gut microbiota composition [45,46,47,48,49]. The interplay between sex hormones, type 2 diabetes, and the gut microbiome is complex and has been increasingly recognized as a factor contributing to interindividual variability in metabolic outcomes. Men and women with diabetes often exhibit differences in gut microbial diversity, structure, and function, which may result from a combination of hormonal, genetic, and lifestyle factors [27,50,51,52,53]. These include age-related increases in inflammation, genomic instability, mitochondrial dysfunction, impaired proteostasis, and epigenetic dysregulation, which contribute to the development of chronic diseases, metabolic disorders, and impaired gut–brain communication.

Our study group consisted of 38 women and 22 men, with a mean age of 64 years and a history of type 2 diabetes lasting for at least 5–10 years. No statistically significant differences were observed in the abundance or taxonomic structure of gut bacteria between women and men. The microbial profiles of the male and female participants largely overlapped, suggesting a similar gut microbiota composition in this elderly diabetic cohort. Physiologically, estrogens have been shown to modulate the gut microbiota by promoting the growth of beneficial bacterial genera, such as *Lactobacillus* and *Bifidobacterium*, reducing inflammation and improving glucose metabolism [52]. Androgens, such as testosterone, may indirectly influence the gut microbiota composition by modulating fat distribution and systemic inflammation, both of which can alter the microbial environment [53].

In our study, sex-specific associations were observed between glycemic markers and the relative abundance of specific bacterial phyla in the gut. Multiple regression analysis revealed a positive correlation between glycemia and the abundance of the phylum *Tenericutes* in male participants, indicating that increases in glycemia were associated with higher levels of *Tenericutes*. This relationship accounted for 67% of the variation in *Tenericutes* abundance, with HbA1c showing a stronger predictive value than fasting glucose (G0′). However, after applying the FDR correction, the association between Tenericutes and G0′ was not statistically significant, whereas the association with HbA1c remained significant. In contrast, in female participants, an inverse correlation was found between glycemia and the abundance of the phylum *Verrucomicrobia*. Specifically, as glycemia increased, the abundance of *Verrucomicrobia*—which includes the beneficial genus *Akkermansia*—decreased, accounting for 58% of the variation observed. This relationship remained significant even after FDR correction, and both G0′ and HbA1c were negatively correlated with *Verrucomicrobia.* The contrasting relationships between the sexes regarding G0′ and bacterial phyla suggest potential sex-specific microbiota interactions with metabolic markers. Males showed a positive correlation with *Tenericutes*, while females showed negative correlations with both *Verrucomicrobia* and OD1 (*Parcubacteria*). For OD1, both G0′ and HbA1c were negatively correlated with OD1 abundance, which remained statistically significant after FDR correction. This may reflect its sensitivity to host metabolic status, glycemic regulation, or shifts in nutrient availability, highlighting its potential involvement in host-microbiome metabolic crosstalk.

Further analysis of the lipid parameters revealed significant associations between the lipid profiles and microbiota composition in female participants. Specifically, TC, HDL-C, and TG levels correlated with *Cyanobacteria*, and HDL-C levels correlated with *Fusobacteria*. These results suggest a potential link between lipid metabolism and the gut microbiota, particularly in females, with significant associations remaining after FDR correction. The significant association between HDL-C and both *Cyanobacteria* and *Fusobacteria* further emphasizes the need for a comprehensive understanding of how lipid profiles interact with specific microbiota communities. It is important to note that although our study identified significant associations, the exact causal mechanisms remain to be elucidated in future studies. Future research should explore the role of microbiome in lipid metabolism through detailed microbial functional analysis and longitudinal studies.

### 4.2. General Microbiota Profile and Microbial Shifts in Patients

Studies on the human gut microbiome have long sought to define the optimal proportions of bacterial phyla associated with good health. Although a definitive consensus has yet to be reached, many authors emphasize that the normal gut flora constitutes a complex bacterial system, typically composed of the following phyla: *Firmicutes* (approximately 64–80%), *Bacteroidetes* (17–23%), *Proteobacteria* (1–8%), and *Actinobacteria* (1–2.5%) in healthy individuals [54,55]. Together, *Firmicutes* and *Bacteroidetes* constitute up to 90% of the gut microbiota, and the *Firmicutes/Bacteroidetes ratio* has been associated with maintaining homeostasis [56]. In healthy individuals, the *F/B ratio* increases significantly with age and is significantly higher in women than in men [57,58,59].

It is estimated that stable ranges of abundance can be determined for about 30% of bacterial species in the gut, forming a universal and relatively stable core microbiome shared by most individuals. The remaining microbial composition is modulated by numerous factors, including physiological processes, age, genetic background, lifestyle (diet and physical activity), pharmacological treatments, and environmental exposures [60,61,62,63].

In our study population, the bacteria constituting the core of the gut microbiota included the most abundant phyla, *Firmicutes* and *Verrucomicrobia*, followed by *Bacteroidetes*, *Proteobacteria*, and *Actinobacteria.* These taxa were present in both males and females. Notably, the *Firmicutes/Bacteroidetes (F/B) ratio* was higher in women (7.92) than in men (5.77), which may reflect sex-related differences in the gut microbiota composition within this population. This suggests that, although subtle compositional differences may exist, the overall structure and richness of the gut microbiota are not strongly sex-dependent in this cohort.

Aging is associated with decreasing levels of sex hormones, which have been shown to influence gut microbial composition. Similar trends were reported by Santos-Marcos et al. [27], who observed a higher relative abundance of *Firmicutes* in premenopausal women than in an age-matched male control group, whereas in postmenopausal women, the *F/B ratio* was similar to that of men. Similarly, data from a Ukrainian cohort showed that women over the age of 50 were significantly more likely than men to exhibit an *F/B ratio* > 1, with this ratio increasing progressively with age [62]. These findings support the notion that both sex- and age-related factors may modulate the gut microbiota composition in individuals with type 2 diabetes.

In diabetic participants in our study, *Firmicutes* were predominantly represented by the order *Clostridiales* (47.8%). The predominance of *Clostridiales*, despite long-standing diabetes and advanced age, may suggest a preserved microbial metabolic capacity in this cohort, possibly influenced by pharmacological or dietary factors. This observation contrasts with previous reports and highlights the need for further investigation of age- and disease-specific microbial profiles.

In contrast, Larsen et al. [63], in a comparative analysis of gut microbiota between patients with diabetes and healthy controls, reported a significantly lower relative abundance of Clostridia in individuals with diabetes (34%) than in controls (53%). This reduction was associated with elevated plasma glucose concentrations. Zhang et al. [64] described the opposite. Although the relatively high abundance of *Clostridia* (approximately 50%) observed in our study suggests preserved intestinal homeostasis in the examined group, this interpretation remains uncertain. Factors such as advanced age, long-standing type 2 diabetes, pharmacotherapy, and sex-related differences in gut microbiota composition (*F/B ratio* in women and men) may influence microbial balance and function, thereby limiting conclusions based solely on taxonomic abundance.

At the phylum level, the gut microbiome of our study population showed a high relative abundance of *Verrucomicrobia* (15.78%). To better assess the balance between dominant bacterial groups, it may be useful to consider the ratio of *Firmicutes* to *Verrucomicrobia (F/V). Verrucomicrobia*, particularly *Akkermansia muciniphila*, plays a crucial role in gut health by maintaining the integrity of the intestinal barrier, modulating host metabolism, and regulating immune responses [65]. Taxonomically, this bacterium is classified as follows: class *Verrucomicrobiae,* order *Verrucomicrobiales,* family *Verrucomicrobiaceae*, and genus *Akkermansia.*

A population-based study conducted in the Netherlands involving 661 women and 474 men reported a higher abundance of *Akkermansia muciniphila* in females, even after adjusting for potential confounding factors such as diet, lifestyle, and medication [66]. A higher abundance of *Verrucomicrobiales (Akkermansia muciniphila)* is generally considered beneficial, as this bacterium has been associated with improved gut barrier function (reduced intestinal permeability), better glucose metabolism (linked to lower insulin resistance), and anti-inflammatory effects (reduced systemic inflammation) [67]. However, in certain metabolic conditions—including type 2 diabetes—excessively high levels of Akkermansia may reflect a compensatory response rather than a direct health benefit.

*Verrucomicrobiae* is considered a beneficial phylum widely distributed in the gastrointestinal tract of healthy individuals, with the capacity to regulate inflammatory processes [68]. Several studies have reported that individuals with type 2 diabetes exhibit a reduced abundance of *Verrucomicrobiae*, a change that is associated with impaired gut barrier function and increased inflammation, potentially contributing to metabolic dysregulation [69].

All patients were treated with metformin, either as a monotherapy or in combination with insulin. As demonstrated in numerous clinical studies [70,71], metformin—a well-established antidiabetic drug used for decades—not only increases the abundance of Akkermansia muciniphila but also promotes the growth of *Escherichia* spp. and *Lactobacillus* spp., while reducing the levels of potentially unfavorable bacteria such as Intestinibacter.

For instance, Cuesta-Zuluaga et al. [58] reported that patients with type 2 diabetes treated with metformin had a 3- to 4-fold higher abundance of *A. muciniphila* in their gut microbiota than those not receiving metformin therapy.

In contrast, a study by Nakajima et al. [72], which enrolled 31 patients with T2DM who were starting metformin treatment for the first time, found no significant differences at the phylum level. These conflicting results may reflect variability in the study design, duration of therapy, baseline microbiota composition, or individual metabolic responses to metformin.

In summary, the gut microbiota of elderly patients with type 2 diabetes in this study was dominated by *Firmicutes*, followed by *Verrucomicrobia* and *Bacteroidetes* phyla. While the *Firmicutes/Bacteroidetes ratio* was higher in women than in men, no statistically significant differences in alpha or beta diversity were observed between the sexes.

### 4.3. Dietary Patterns and Their Relationship with Gut Microbiota in Elderly Diabetic Patients

Diet plays a pivotal role in shaping the gut microbiota composition and function, especially in individuals with type 2 diabetes [73,74,75,76,77]. Analysis of the 3-day dietary records revealed that men had significantly higher intakes of total energy, protein, saturated fatty acids, and carbohydrates than women. These sex-related dietary differences are consistent with the known physiological and metabolic requirements, as men generally have greater energy needs.

A diet rich in animal proteins (red meat and dairy products) could increase the abundance of *Bacteroides, Alistipes,* and *Bilophila*, leading to an increase in trimethylamine oxide (TMAO) (ammonia, nitrosamines, and trimethylamine N-oxide), a compound known for its proatherogenic potential, playing a role in cardiovascular diseases [78]. In recent years, legumes (mainly lentils, beans, chickpeas, and peas) have received increasing attention as a sustainable source of plant protein compared to animal protein. Indeed, the consumption of legumes is associated with positive gut microbial changes in rodents and humans, with improved growth of the genera *Bifidobacterium, Faecalibacterium, Clostridium, Eubacterium*, and *Roseburia*, which are the primary producers of butyrate and acetate [78]. In our senior group, the primary source of protein was mainly meat dishes, both among men and women. According to Malesza et al. [79], Zheng et al. [80], a high-fat diet (HFD)—specifically saturated fatty acids–leads to dysbiosis with a decrease in *Bacteroidetes* and an increase in *Firmicutes* and *Proteobacteria*, resulting in increased insulin resistance, gut permeability, and adipose tissue inflammation. Similar results regarding changes in *Firmicutes* and *Bacteroides* abundance were observed in both sexes in our study.

In contrast, plant-based diets rich in fiber and complex carbohydrates are known to support the growth of *Bacteroidetes* and *Verrucomicrobia*, including beneficial taxa such as *Akkermansia muciniphila*. In our cohort, fiber intake did not differ significantly between the sexes, suggesting that the observed sex-related microbial differences, such as the higher *F/B ratio* in women are unlikely to be attributed to differences in fiber consumption.

Although statistically significant differences in total energy and macronutrient intake were observed between men and women in our study, the relative contribution of macronutrients to energy intake and fiber consumption did not differ substantially between the sexes. These findings are consistent with the current literature, suggesting that sex-related differences in gut microbiota composition among older adults with type 2 diabetes may be minimal when dietary quality is similar.

### 4.4. Limitations of the Study

The present study had several limitations. First, the number of older adult patients with type 2 diabetes included in the analysis was relatively small. Therefore, the findings should be considered preliminary and specific to the cohort studied. Although we applied FDR correction to account for multiple comparisons, the sample size remains a limitation, and further studies with larger cohorts are needed to confirm these results. These observations, especially the sex-dependent associations, may serve as a basis for hypothesis generation in larger confirmatory studies.

Another limitation is the lack of a younger control group and the absence of comparison across different antidiabetic treatment strategies. Including younger individuals (e.g., aged 40–50 years) and patients treated with other antihyperglycemic agents could help differentiate age-related microbiota changes from those linked to glycemic control or medication effects.

In addition, a broader issue in microbiome research is the lack of methodological standardization, as emphasized by Mogna-Peláez et al. [47]. Variations in DNA extraction protocols, sequencing platforms, and bioinformatics pipelines make cross-study comparisons challenging. Furthermore, the limited functional characterization of many microbial taxa—some of which were detected in our analysis—complicates the biological interpretation of the results.

## 5. Conclusions

In this cohort of elderly patients with type 2 diabetes, no statistically significant differences were observed in the overall composition or diversity of the gut microbiota between women and men. However, sex-specific associations were identified between glycemic markers and the abundance of specific bacterial phyla. In men, HbA1c was positively associated with the abundance of *Tenericutes*, while in women, both G0′ and HbA1c were negatively correlated with *Verrucomicrobia* and ODI (*Parcubacteria)*. The contrasting relationships between genders regarding G0′ and bacterial phyla suggest potential gender-specific microbiota interactions with metabolic markers. Increased glycemia was associated with a lower abundance of *Verrucomicrobia*, including the beneficial genus *Akkermansia*. Additionally, a high relative abundance of *Clostridiales* was observed in the study population despite the presence of long-standing diabetes.

These findings highlight the importance of considering sex-specific microbiota–metabolism interactions in older adults with type 2 diabetes. Further research is needed to explore the functional implications of these associations and their potential relevance to metabolic health and therapy.

In elderly patients with type 2 diabetes, our findings suggest that gut microbiota composition is more closely related to glycemic status than to sex. While women exhibited a higher *Firmicutes/Bacteroidetes* ratio than men, no significant differences were observed in alpha or beta diversity, suggesting that the microbial community structure and richness were broadly comparable between the sexes.

These observations reinforce the value of integrating sex-specific perspectives into microbiome research, particularly in the context of age-related metabolic diseases.

## Figures and Tables

**Table 1:** Study group characteristics (the results are expressed as median and interquartile range).

Parameter	All Patients	Females (F)	Males (M)	*p*
	N = 60	N = 38	N = 22	
Age [years]	64.0 (59.5–72.0)	69.5 (61.0–75.0)	60.0 (55.0–63.0)	0.004
G0 [mmol/L]	7.3 (6.2–9.7)	6.7 (5.9–10.4)	7.9 (6.6–9.2)	NS
HbA1c [%]	7.2 (6.2–8.7)	7.0 (6.2–8.9)	7.4 (6.2–8.5)	NS
TC [mg/dL]	179.7 (147.4–199.8)	186.8 (158.2–205.8)	172.6 (143.6–192.6)	NS
HDL [mg/dL]	48.4 (38.2–58.0)	52.8 (41.4–66.6)	37.7 (32.9–51.4)	0.0005
LDL [mg/dL]	95.7 (67.1–120.8)	94.6 (68.2–123.0)	95.7 (65.3–118.7)	NS
Non-HDL [mg/dL]	125.7 (93.8–148.2)	125.7 (88.6–148.7)	125.8 (94.2–146.4)	NS
TAG [mg/dL]	141.8 (117.3–184.0)	134.7 (104.8–183.4)	144.7 (133.7–202.0)	NS
Cre [umol/L]	68.0 (58.8–89.9)	63.7 (54.7–79.2)	74.8 (62.0–93.1)	NS

NS—not significant Under the table.

**Table 2:** Analysis of 3-day dietary records in the study group.

Parameter	All Patients (Mean ± SD)	Women (Mean ± SD)	Men (Mean ± SD)	*p*-Value
Energy (kJ)	8457.1 ± 824	8087.9 ± 824	8960.7 ± 796	0.000125
Energy (kcal)	2013.6 ± 297	1925.7 ± 297	2133.5 ± 307	0.005937
Protein (g)	72.9 ± 9.6	68.6 ± 9.6	79.4 ± 9.8	0.000055
% Energy from Protein	14.5 ± 2.7	14.3 ± 2.7	14.9 ± 2.9	0.386249
Fat (g)	71.2 ± 11.3	68.4 ± 10.8	75.6 ± 12.3	0.028
% Energy from Fat	31.7 ± 14.2	31.9 ± 15.6	33.9 ± 13.6	0.635
Saturated Fatty Acids (g)	30.3 ± 12.4	25.1 ± 11.6	36.1 ± 13.1	0.001073
Monounsaturated Fatty Acids (g)	31.1 ± 10.9	24.6 ± 10.3	34.7 ± 11.7	0.001073
Carbohydrates (g)	265.1 ± 74	244.4 ± 86	299.7 ± 71	0.008693
% Energy from Carbohydrates	52.8 ± 12.3	52.8 ± 12.6	54.2 ± 11.6	0.41856
Dietary Fiber (g)	22.9 ± 5.9	22.6 ± 6.7	23.7 ± 5.8	0.512300
Vitamin A [µg]	1348.2 ± 768	1468.5 ± 845	1157.6 ± 729	0.0139
Vitamin B6 [mg]	1.64 ± 0.72	1.42 ± 0.86	1.70 ± 0.57	0.136
Vitamin D [µg]	4.05 ± 2.3	3.90 ± 2.2	4.78 ± 2.5	0.178
Vitamin E [mg]	11.6 ± 3.6	9.98 ± 3.8	12.5 ± 3.4	0.109
Vitamin C [mg]	91.9 ± 52	89.7 ± 57	94.2 ± 45	0.138

SD—Standard Deviation.

## Data Availability

The data that support the findings of this study are available from the corresponding author upon reasonable request.
